# Addressing the Detection of Ammonium Ion in Environmental Water Samples via Tandem Potentiometry–Ion Chromatography

**DOI:** 10.1021/acsmeasuresciau.1c00056

**Published:** 2022-01-20

**Authors:** Renato L. Gil, Célia G. Amorim, Maria Cuartero

**Affiliations:** † LAQV-REQUIMTE, Department of Chemical Sciences, Faculty of Pharmacy, University of Porto, Jorge Viterbo Ferreira, 228, 4050-313 Porto, Portugal; ‡ Department of Chemistry, School of Engineering Sciences in Chemistry, Biotechnology and Health, KTH Royal Institute of Technology, Teknikringen 30, SE-100 44 Stockholm, Sweden

**Keywords:** ammonium, potentiometry, ion-selective electrodes, ion chromatography, environmental water samples, tandem analytical technique

## Abstract

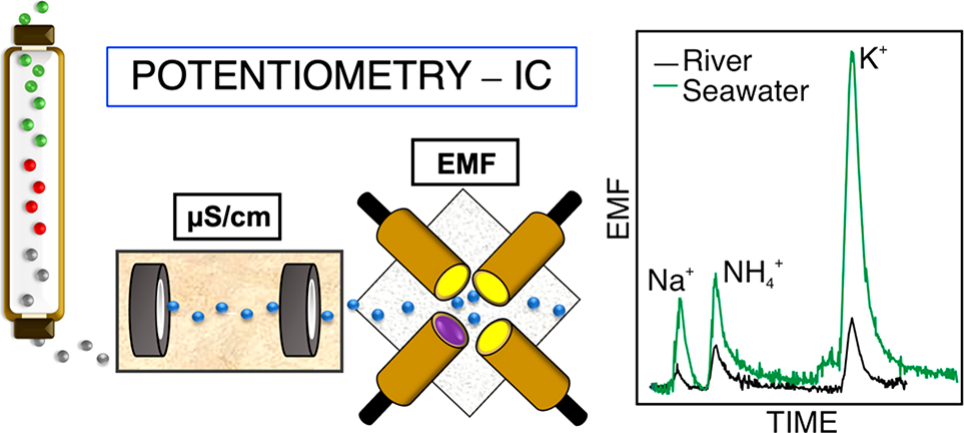

An analytical methodology for detecting ammonium ion (NH_4_^+^) in environmental water through potentiometry–ion chromatography (IC) in tandem is presented here. A multielectrode flow cell is implemented as a potentiometric detector after chromatographic separation of cations in the sample. The electrodes are fabricated via miniaturized all-solid-state configuration, using a nonactin-based plasticized polymeric membrane as the sensing element. The overall analytical setup is based on an injection valve, column, traditional conductometric detector, and new potentiometric detector (in that order), permitting the characterization of the analytical performance of the potentiometric detector while validating the results. The limit of detection was found to be *ca*. 3 × 10^–7^ M NH_4_^+^ concentration after linearization of the potentiometric response, and intra- and interelectrode variations of <10% were observed. Importantly, interference from other cations was suppressed in the tandem potentiometry–IC, and thus, the NH_4_^+^ content in fresh- and seawater samples from different locations was successfully analyzed. This analytical technology demonstrated a great potential for the reliable monitoring of NH_4_^+^ at micromolar levels, in contrast to the conductivity detector and previously reported NH_4_^+^ potentiometric sensors functioning in batch mode or even coupled with IC. Additionally, the suitability of the potentiometric cell for selective multi-ion analysis in the same sample, *i.e.*, Na^+^, NH_4_^+^, and K^+^ in water, has been proven.

The ammonium ion (NH_4_^+^) is one of the primary compounds involved in the biogeochemical cycle of nitrogen (N) in aquatic environments.^1,2^ Despite its distribution in freshwaters being highly variable regionally, seasonally, and spatially within streams and lakes, the concentration of total ammonia nitrogen (TAN) in well-oxygenated waters is usually low (typical values ranging from 7 to 60 μg TAN L^–1^).^1^ When the N-cycle becomes unbalanced, detrimental consequences manifest for the water life: high NH_4_^+^ levels (>2 mg TAN L^–1^) are known to translate into undesired eutrophication and poor water quality.^1,2^ Excessive NH_4_^+^ levels may occur through direct means (municipal effluent discharges and animal waste) or indirect sources (nitrogen fixation, air deposition, and runoff from agricultural lands).^3^ Altogether, NH_4_^+^ content is considered an important environmental indicator, and monitoring it is crucial for effective water ecosystem preservation.^2^

Analytical methodologies for NH_4_^+^ detection have been developed for many years.^4,5^ Spectrophotometric, conductometric, and potentiometric readouts stand out for the specific application of water analysis among the available techniques. Potentiometric ion-selective electrodes (ISEs), based on ion-selective membranes (ISMs), are particularly interesting because of their capacity for decentralized measurements.^6^ Indeed, a recent review has thoroughly discussed the analytical features and applications of NH_4_^+^-selective electrodes reported in the past decade.^7^ Considering the ionophore (or receptor) as the core element of the ISM, effective NH_4_^+^ recognition has been accomplished over many years.^7^ At present, nonactin is the most utilized ionophore, despite its significant drawback of forming complexes with other cations of similar ionic size and charge to NH_4_^+^, such as potassium (K^+^) and sodium (Na^+^) ions. Thus, the presence of excess K^+^ and Na^+^ compared to NH_4_^+^ in the sample could hinder accurate determination with ISEs, rendering the effective detection of NH_4_^+^ difficult in either aqueous or other samples. To overcome this issue of selectivity, several strategies have been proposed in the literature aside from the search for more selective ionophores.^7^

Athavale et al. reported a mathematical treatment to correct the recorded ISE potential, which considered the K^+^ levels in freshwater together with the NH_4_^+^/K^+^ selectivity coefficient.^8^ Briefly, all-solid-state ISEs were integrated into a submersible device, and the mathematical treatment was used to measure the NH_4_^+^ in lakes. While this approach provided accurate profiles from the depth where the NH_4_^+^ concentration was greater than 10 μM, the detection of lower concentrations remained unfeasible. Similarly, potentiometric electronic tongues for NH_4_^+^ detection have been proposed, which again require the previous determination of the selectivity coefficients. The Diamond and del Valle groups have targeted synthetic water samples, as well as river water and wastewater with this approach, claiming accurate measurements only at the millimolar level.^9,10^ Speciation of N compounds has also been pursued with electronic tongues.^11^ Despite very valuable efforts reported in the literature until now, demonstrating the utility of a statistical analysis of potentiometric data (especially in the direction of sample classification),^12−15^ the accurate quantification of NH_4_^+^ still remains a challenge in environmental water samples.

Some very interesting analytical concepts have been developed to reduce or eliminate interference(s) in the sample before applying potentiometric NH_4_^+^ detection.^7^ Such strategies have been applied to clinical samples rather than environmental water, e.g., the method of indirect creatinine determination reported by Liu et al.^16^ All-solid-state ISEs for NH_4_^+^ were modified with the creatinine deiminase enzyme on top of the ISM and an outer anion-exchange membrane, which prevents the cations passing from the sample to the core of the sensing element (the ISM with the enzyme).^16^ Hence, the potentiometric response represents only the NH_4_^+^ formed in the quantitative enzymatic creatinine reaction, without any K^+^/Na^+^ interference. Effectively, separation science may offer suitable opportunities for NH_4_^+^ detection in the form of a broad portfolio of techniques to separate ions in the complex matrix via differences in affinity.^17^

This paper presents an analytical methodology for accurately determining NH_4_^+^ in environmental water samples based on tandem potentiometry–ion chromatography (IC). A multielectrode flow cell containing three miniaturized all-solid-state ISEs functionalized with nonactin as the ionophore is proposed as the detector. The cell is coupled in-line with an IC system based on a cation-exchange column. In addition, a traditional conductivity detector is implemented, which allows the validation of this new methodology. Given the selectivity limitations generally presented by nonactin-based ISEs, the column is exploited to provide the unequivocal separation of NH_4_^+^ from the main interfering ions (K^+^ and Na^+^) in real water samples. In addition, the potentiometric cell is explored for multication detection in environmental water samples.

## Experimental Section

### Preparation of the Ion-Selective Electrodes and Reference Electrode

**Figure 1:**
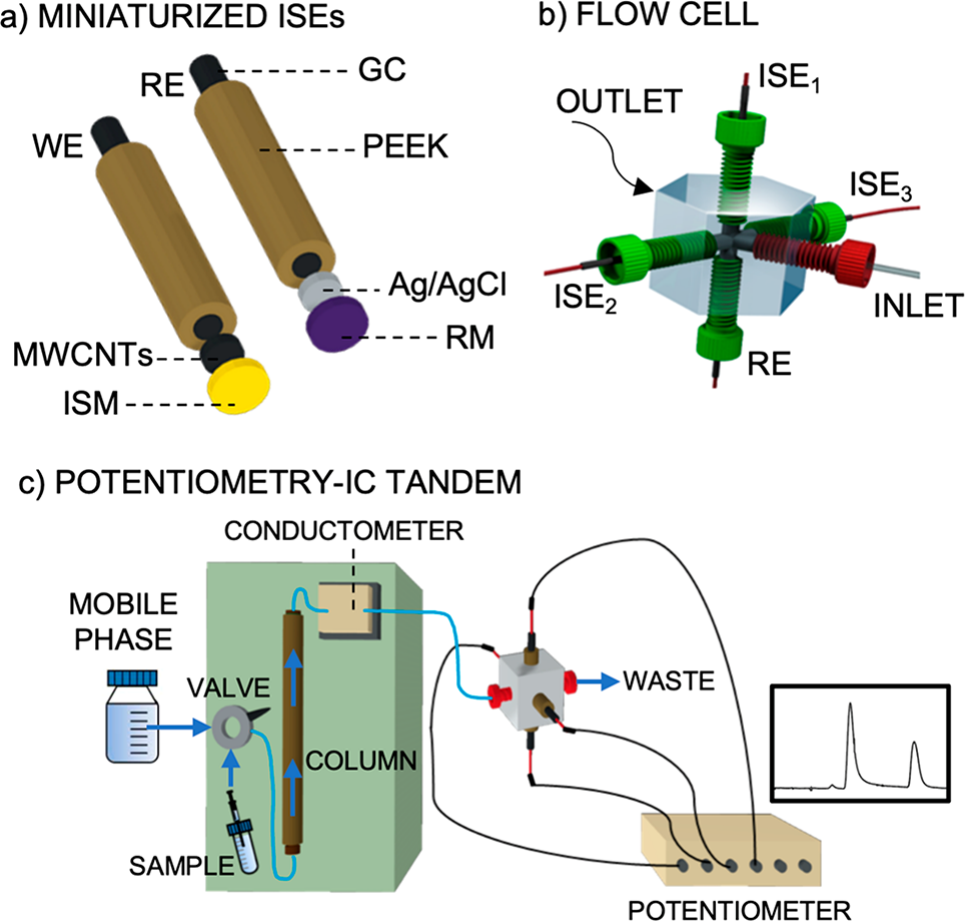
(a) Miniaturized ISEs based on glassy carbon (GC). The working electrode (WE) was prepared with MWCNTs and the ISM. The reference electrode (RE) was prepared with Ag/AgCl ink and the RM on top. (b) Multielectrode flow cell with three ISEs and the RE, the inlet, and the outlet. (c) Tandem potentiometry–IC: the sample is injected through the valve and carried by the mobile phase through the chromatographic column.

Miniaturized handmade glassy carbon electrode bodies were fabricated as detailed in the Supporting Information.^18^ All-solid-state ISEs for NH_4_^+^, K^+^, and Na^+^ were prepared via the functionalization of the glassy carbon electrodes with the ion-to-electron transducer (multiwalled carbon nanotubes, MWCNTs) and ISMs as follows (Figure 1a). Membrane cocktails containing a polymeric matrix, plasticizer, cation exchanger, and the corresponding ionophore dissolved in tetrahydrofuran (THF) were prepared to fabricate NH_4_^+^-, K^+^-, and Na^+^-selective electrodes, as detailed in the Supporting Information. The surface of the glassy carbon electrode was first modified by drop casting 4 × 5 μL of 1 mg mL^–1^ MWCNT solution in ethanol.^16^ Each layer was allowed to dry for 10 min before the next layer was added. Then, the ISM was formed by drop casting 4 × 5 μL of the corresponding membrane cocktail on top of the MWCNT film. Each layer was allowed to dry for 20 min. Finally, the electrodes were conditioned at least overnight in a 10^–3^ mol L^–1^ solution of the respective cation analyte.

The reference electrode was fabricated by modifying the surface of the glassy carbon electrode with a silver/silver chloride (Ag/AgCl) ink (C21310007D3, GWENT Group, UK),^16^ followed by oven-curing at 100 °C for 10 min. The reference membrane (RM, composition in the Supporting Information) was drop casted on the Ag/AgCl coating (4 × 5 μL); each layer was dried for 10 min before depositing the next one. The reference electrode was left to dry overnight and conditioned in 3 M KCl for at least 48 h. The electrode was stored in a 3 M KCl solution when not in use.

### Preparation of the Multielectrode Potentiometric Flow Cell

Three miniaturized ISEs were inserted in the multielectrode flow cell together with the reference electrode.^18^ The flow cell was a cube made of an acrylic block with six drilled holes: two on opposite sides for the inlet and outlet, one for the reference electrode, and three others to host the ISEs (Figure 1b). The electrodes were incorporated using a plastic screw (flangeless yellow ferrule and a male green nut, 1/8, IDEX Health & Science). The electrical connection was made outside the cube with small crocodile clamps connected to each electrode. For the inlet and outlet, a blue ferrule and a male red nut (1/16, IDEX Health & Science) were used to connect the PTFE tubing.

### Potentiometry–Ion Chromatography System

The potentiometry–IC tandem measurements (Figure 1c) were conducted using an 850 Professional IC system (Metrohm Nordic) combined with a high-pressure pump and a six-port high-pressure injection valve. A guard-column (Metrosep C6 Guard/4.0; 5 × 2.0 mm I.D., 5 μm; Metrohm, Switzerland) was placed before the analytical cation-exchange column (Metrosep C6; 150 × 4.6 mm I.D., 4 μm, Metrohm, Switzerland). The outlet of the cation-exchange column was connected to the Metrohm conductivity detector. MagicNet chromatography data system software (Metrohm, Switzerland) was used to control the IC components and for data processing in the conductivity detector. The multielectrode flow cell was coupled in-line with the outlet of the conductivity detector using PEEK tubing (L × O.D. × I.D. = 300 mm × 1/16 in. × 0.25 mm, Metrohm) introduced in the ferrule and nut of the inlet of the cell.

The mobile phase used for the potentiometric measurements was 2.5 × 10^–3^ mol L^–1^, as recommended by the column manufacturer for a proper functioning and ions’ separation. Notably, different batches of the mobile phase solution were found to provide slightly different retention times for analogous ion peaks in the same sample. The injection volume and flow rate were optimized to be 10 μL and 0.9 mL min^–1^, respectively.

## Results and Discussion

### Analytical Evaluation of Miniaturized Potentiometric Sensors for Ammonium

**Figure 2:**
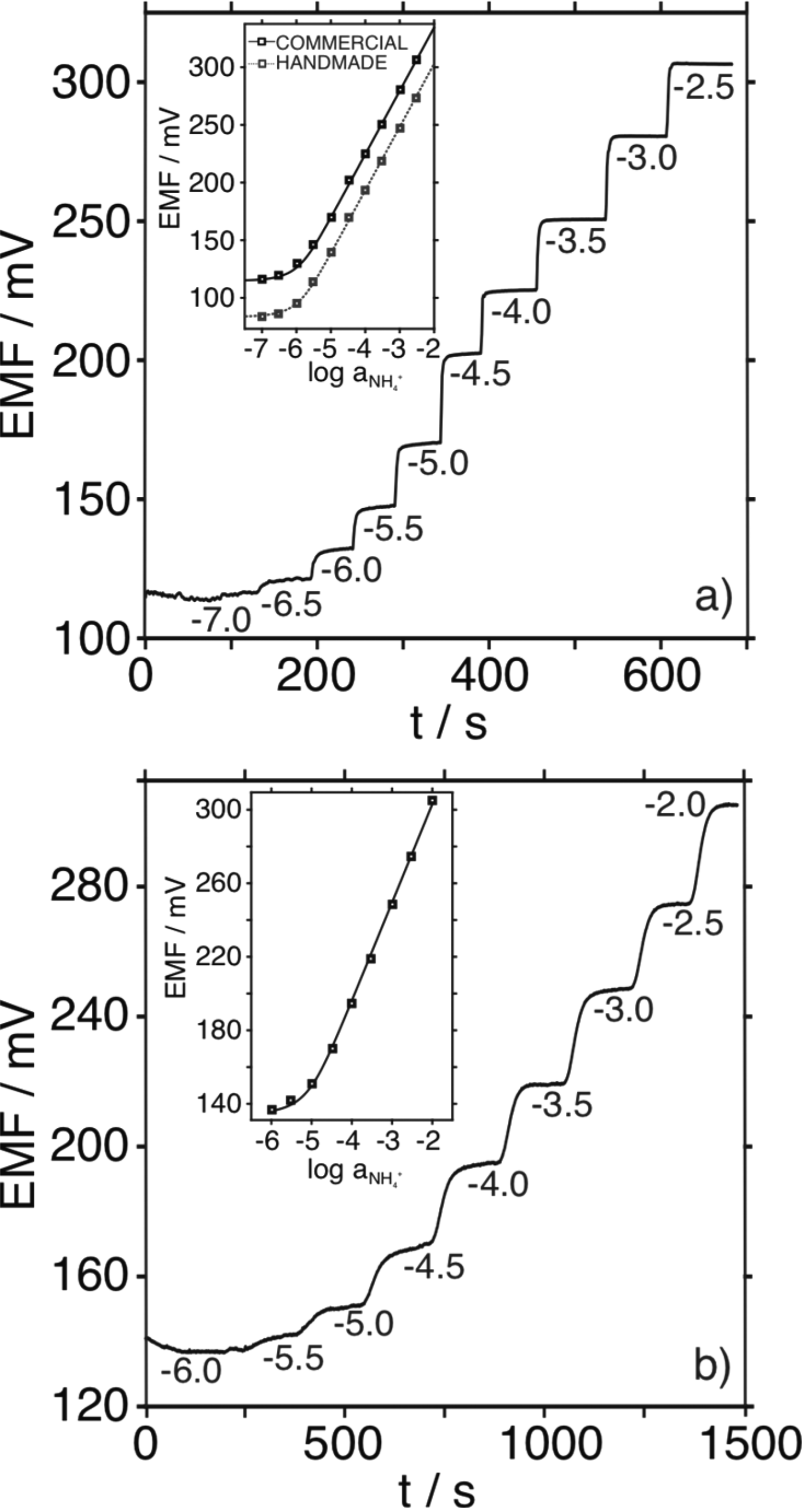
(a) Dynamic response of one NH_4_^+^-selective electrode in batch mode at increasing NH_4_^+^ activity and using the commercial Ag/AgCl reference electrode. Inset: Corresponding calibration plot and that obtained with the handmade reference electrode. (b) Response of one NH_4_^+^-selective electrode in the flow cell (0.5 mL min^–1^). Inset: Corresponding calibration graph.

The analytical performance of the NH_4_^+^-selective electrodes was first evaluated under batch conditions (background electrolyte: 2.5 × 10^–3^ mol L^–1^ nitric acid) against the commercial Ag/AgCl reference electrode. Figure 2a shows the dynamic potentiometric response of the NH_4_^+^-selective electrode at increasing activities of NH_4_^+^. Fitting the logarithmic NH_4_^+^ activity versus the corresponding steady-state potential to the Nernst equation (inset of Figure 2a) revealed that the electrode followed a Nernstian behavior, with a slope of 54.6 mV dec^–1^, with a linear range of response (LRR) from 3.0 × 10^–6^ to 3.0 × 10^–3^ NH_4_^+^ activity and a limit of detection (LOD) of 6.1 × 10^–7^ NH_4_^+^ activity. The response time (t_95_)^19^ was rapid, from 2.3 to 2.9 s within the LRR. Furthermore, the response was found to be very reproducible considering subsequent calibrations for the same electrode (slope of 54.1 ± 0.9 mV dec^–1^ and intercept of 447.0 ± 2.3 mV, *n* = 3) and equally prepared electrodes (slope of 54.5 ± 1.8 mV dec^–1^ and intercept of 439.9 ± 9.4 mV, *n* = 3), with variation coefficients for the calibration parameters of <4%. Analogous calibration parameters, but with a slight shift in the offset potential, were achieved with the miniaturized handmade reference electrode (dotted line in Figure 2a): slope of 55.5 ± 0.7 mV dec^–1^, LRR from 3.0 × 10^–6^ to 3.0 × 10^–3^ NH_4_^+^ activity and LOD of 2.0 ± 0.5 × 10^–7^ NH_4_^+^ activity (*n* = 3 electrodes).

Three NH_4_^+^-selective electrodes were inserted in the microfluidic cell with the handmade reference electrode (Figure 1b) for characterization under flow-mode conditions using a peristaltic pump (0.5 mL min^–1^, 2.5 × 10^–3^ mol L^–1^ nitric acid background). Figure 2b displays the dynamic response at increasing NH_4_^+^ activities and the calibration graph corresponding to one of the three NH_4_^+^-selective electrodes as an example. The average calibration parameters of the three electrodes were a slope of 52.5 ± 1.2 mV dec^–1^, LRR from 3.0 × 10^–5^ to 1.0 × 10^–2^ NH_4_^+^ activity, an intercept of 410.0 ± 20.3 mV, and a LOD of (7.5 ± 0.2) × 10^–6^ NH_4_^+^ activity. A slightly lower slope was found, with the LRR starting from an NH_4_^+^ activity 1 order of magnitude higher than in the batch mode. The LOD was also 1 order of magnitude higher in the flow mode. Overall, the response was slightly worse in the microfluidic cell, likely due to the flow-cell configuration *per se* (tangential mode) and the hydrodynamic conditions of the measurements. Other authors found that the tangential mode hindered achieving approximately 95% of the steady-state signal, especially in low ion analyte activities. In addition, relatively low flow rates (such as 0.5 mL min^–1^) do not propitiate the removal of infinitesimal concentrations at the membrane surface, with the result that low analyte concentrations require longer times to attain the steady-state signal.^20^

Potentiometric selectivity coefficients were determined for Mg^2+^, Ca^2+^, Li^+^, Na^+^, and K^+^ ions using the well-known separate solution method.^21^ Notably, the apparent values were calculated since this method assumes the slope in the calibration graph of the interfering cation equal to that of the primary one, which was not the case. In addition, the interfering cations were tested in order of their lipophilicity and with NH_4_^+^ (the most preferred cation) in the last place to avoid bias in the values.^21^ The potentials provided by 10^–3^ activity of each interfering cation and for NH_4_^+^ were used to calculate the apparent logarithmic selectivity coefficients: log *K*_NH_4_^+^,X_^pot^ = −4.15 ± 0.22, −4.30 ± 0.16, −2.50 ± 0.12, −2.20 ± 0.28, and −0.98 ± 0.12 for X = Mg^2+^, Ca^2+^, Li^+^, Na^+^, and K^+^ (*n* = 3), respectively. The K^+^ ion was found to give the strongest interference, showing a similar value of the logarithmic selectivity coefficient to those previously reported for ISEs containing nonactin as the ionophore (from −0.42 to −1.8).^7^

Considering typical K^+^ concentrations in freshwater and seawater (0.1 and 10 mM, respectively) together with the calculated logarithmic coefficient, the minimum LODs that could be reached for NH_4_^+^ are estimated to be *ca*. 10^–5^ and 10^–3^ M (calculated as [*K*_NH_4_^+^,K^+^_^pot^·*c*_K^+^_]). As a result, the potentiometric cell developed in this work is unsuitable for seawater analysis and can only be applied to freshwater samples with NH_4_^+^ exceeding 10^–5^ M, *i.e.*, heavily contaminated samples. To overcome this limitation, the tandem potentiometric–IC is following investigated, wherein the effective separation of NH_4_^+^ from any interfering cations present is expected.

### Coupling the Potentiometric Cell with the Ion Chromatography System

**Figure 3:**
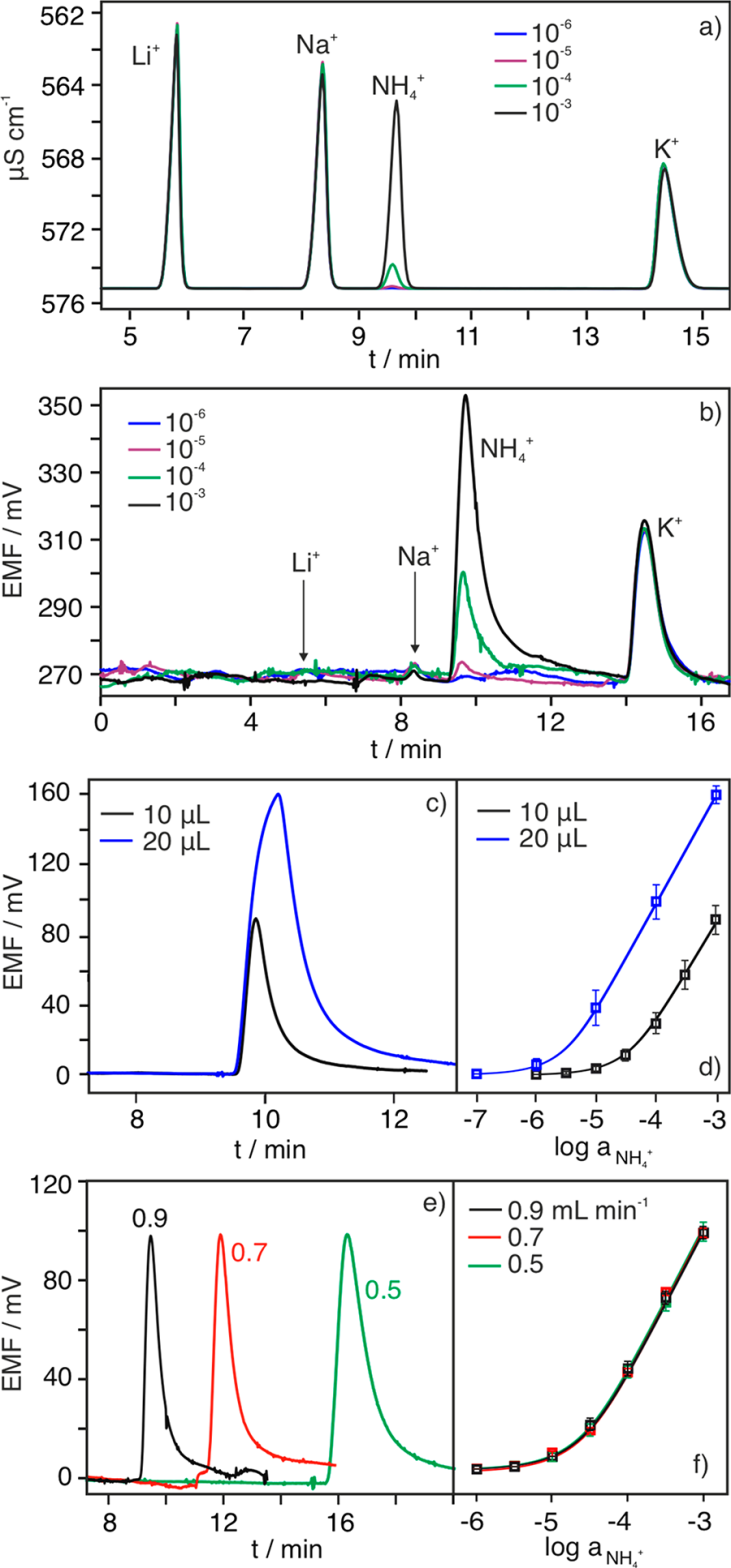
(a) Conductivity chromatograms at increasing NH_4_^+^ activities and 1 × 10^–3^ activity of the other cations (10 μL volume, 0.9 mL min^–1^). (b) Potentiometric chromatograms at increasing NH_4_^+^ activity and 1 × 10^–3^ activity of the rest of the cations (10 μL volume, 0.9 mL min^–1^). (c) Potentiometric chromatograms with 10 and 20 μL injected volume of 1 × 10^–3^ NH_4_^+^ activity (0.9 mL min^–1^). (d) Averaged calibration graphs (*n* = 3). (e) Potentiometric chromatograms with 1 × 10^–3^ NH_4_^+^ activity at 0.5, 0.7, and 0.9 mL min^–1^ (10 μL volume). (f) Averaged calibration graphs (*n* = 3). Background: 2.5 × 10^–3^ mol L^–1^ nitric acid.

The separation capability of the proposed tandem potentiometry–IC was first evaluated with a mixture containing equal activities (1.0 × 10^–3^) of Li^+^, Na^+^, K^+^, and NH_4_^+^. The black lines in Figure 3a and b indicate the chromatograms observed with both the conductivity detector and one of the NH_4_^+^-selective electrodes in the potentiometric cell (sample volume of 10 μL, flow rate of 0.9 mL min^–1^) for the mentioned mixture. Peak identification was performed from separate solutions of each cation (data not shown).

The order of elution for the cations was Li^+^ (5.8 min), Na^+^ (8.4 min), NH_4_^+^ (9.6 min), and K^+^ (14.4 min), with the retention times being *ca*. 4 s longer for the potentiometric detector than for the conductivity detector, because of their position in the experimental setup. While the chromatogram provided by the conductivity detector yields similar peak areas for the four cations, the chromatogram provided by the potentiometric cell displayed a higher potential readout for NH_4_^+^ than for the other cations (Table S1). Indeed, the peaks for Li^+^ and Na^+^ were very weak. Furthermore, when the NH_4_^+^ content in the sample was decreased to 1.0 × 10^–6^ activity, while maintaining the levels for the rest of the cations (1.0 × 10^–3^ activity), the peaks for Li^+^, Na^+^, and K^+^ did not change. However, the peak height for NH_4_^+^ decreased in both the potentiometric and conductivity chromatograms (Figure 3a and b and Table S2).

Then, the peak for the 1 × 10^–6^ NH_4_^+^ activity was well-observed with the potentiometric detector, but it was difficult to be identified and/or quantified by the conductivity detector. Notably, in the case of the potentiometric detector, the returning to the base signal in the NH_4_^+^ peak appears to be slower for increasing activity, which seems to not disturb the K^+^ signal, as demonstrated with the overlapping K^+^ peaks in Figure 3b. Importantly, this behavior is not expected to influence the accuracy of the NH_4_^+^ analysis even at high activity/concentrations (around mM), since the signal does return to the initial baseline at the end of the chromatogram.

Next, the effect of the injected sample volume and flow rate on the chromatograms was investigated. For this purpose, samples with increasing activity of NH_4_^+^ (from 1 × 10^–6^ to 1 × 10^–3^, *n* = 3) were analyzed using two different sample volumes (10 and 20 μL, flow rate of 0.9 mL min^–1^) and three flow rates (0.5, 0.7, and 0.9 mL min^–1^, sample volume of 10 μL). Figure 3c–f compare the chromatograms observed with the same electrode at 1 × 10^–3^ NH_4_^+^ activity under different volume and flow rate conditions, together with the averaged calibration graphs (*n* = 3) at increasing NH_4_^+^ activity.

In the case of the injected sample volume, and considering a 10^–3^ NH_4_^+^ activity (Figure 3c), the peak was higher (81.7 mV versus 158.0 mV) and wider (time difference at 5% of the peak maximum of 2.4 min versus 4.2 min) when the volume was increased from 10 to 20 μL. Also, the retention time was slightly longer (9.8 min versus 10.3 min). This trend appeared for all tested NH_4_^+^ activities (Figure S1). In principle, an increase in peak height, width, and retention time is expected with increasing sample volume as the analyte plug injected into the detector increases in length. Consequently, the separation efficiency was slightly better with the 10 μL sample (Table S3: number of plates of 2544 versus 1146 for NH_4_^+^ activity > 10^–5^). However, the average calibration graph (*n* = 3) with a 20 μL sample volume displayed a hyper-Nernstian slope (62.1 mV versus 57.3 mV for 20 and 10 μL, respectively, Figure 3d) as well as a lower LOD (1 × 10^–7^ versus 6 × 10^–6^ NH_4_^+^ activity, Figure 3d). The peaks at lower NH_4_^+^ activities appear slightly more distinct with the 20 μL volume than the 10 μL volume (Figure S1).

Concerning the flow rate, we found increasing retention times (9.5, 11.9, and 16.4 min) and widths (1.7, 2.2, and 2.9 min) for the NH_4_^+^ peak with decreasing flow rate (Figure 3e), corresponding to a decrease in the separation efficiency (number of theoretical plates 1584, 2254, and 3040 for 0.5, 0.7, and 0.9 mL min^–1^, respectively, Table S4). Nevertheless, we did not find any significant difference in the averaged calibration parameters (*n* = 3) of the different flow rates (Figure 3f; the corresponding chromatograms are provided in Figure S2). Given these results, a sample volume of 10 μL and a flow rate of 0.9 mL min^–1^ were selected for further experiments as a compromise between separation efficiency, calibration parameters, and analysis time.

### Analytical Performance of the Tandem Potentiometry–Ion Chromatography

**Figure 4:**
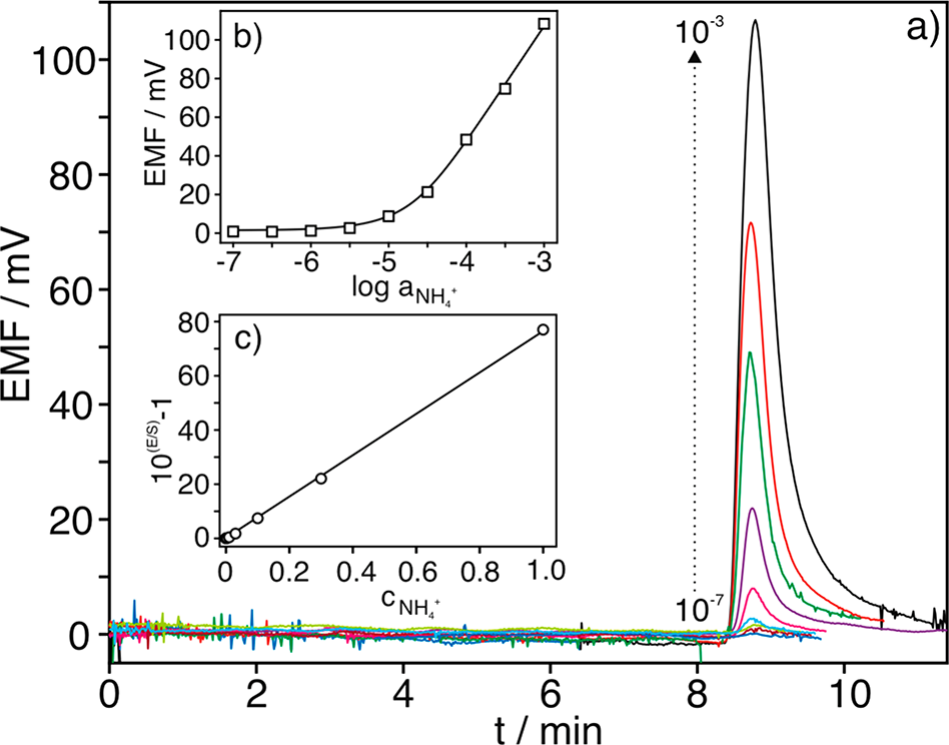
(a) Chromatograms at increasing NH_4_^+^ activity. (b) Corresponding averaged calibration graph (*n* = 3). (c) Linearization of the averaged calibration graph (2.5 × 10^–3^ mol L^–1^ nitric acid, 10 μL sample volume, flow rate of 0.9 mL min^–1^).

The sensitivity, LRR, LOD, limit of quantification (LOQ), and reproducibility were investigated at the optimized experimental conditions. Potentiometric chromatograms at increasing NH_4_^+^ activity (10^–7^–10^–3^) are presented in Figure 4a. The averaged calibration graph for the potentiometric cell (*n* = 3 electrodes) revealed a slope of 59.5 ± 1.2 mV dec^–1^ within the LRR (from 3.0 × 10^–5^ to 10^–3^ NH_4_^+^ activity) and a LOD of 2.5 × 10^–6^ (Figure 4b). Pursuing the detection of lower NH_4_^+^ levels, we investigated a transformation for the calibration graph via linearization of the entire logarithmic response.^22^ We used the linear fitting of the (10^*E*/*S*^–1) magnitude (where *E* refers to the potential and *S* to the slope in the LRR when plotting the potential versus log *a*_*i*_) against the NH_4_^+^ concentration (Figure 4c), as previously reported for other chromatographic–potentiometric tandem systems.^22^

Following this approach, an expanded LRR from 10^–6^ to 10^–3^ mol L^–1^ NH_4_^+^ with a sensitivity of 76.6 ± 2.2 L mmol ^–1^ (*R*^2^ = 0.9986) was found. The LOD and LOQ were calculated as the concentration corresponding to a signal-to-noise ratio of three and ten times, respectively, yielding values of 3.0 × 10^–7^ and 1.0 × 10^–6^ mol L^–1^ NH_4_^+^. Under the same experimental conditions, the LOD and LOQ with the conductivity detector were calculated as 1.0 × 10^–6^ and 3.0 × 10^–6^ mol L^–1^ (Figure S3). The reproducibility was evaluated by the triplicate injection of 3.0 × 10^–6^, 1.0 × 10^–5^, and 3.0 × 10^–4^ mol L^–1^ NH_4_^+^ measured with three NH_4_^+^-selective electrodes and the injection of 1.0 × 10^–5^ mol L^–1^ NH_4_^+^ over four consecutive days. Intraelectrode reproducibility for the peak potentials revealed a variation coefficient < 5%. Interelectrode reproducibility showed a variation coefficient of <9%. Finally, variations of *ca*. 0.5% and 7% were observed for the calibration parameters of the NH_4_^+^-selective electrodes in the second and fourth days of their continuous usage, respectively.

### Analysis of Natural Water Samples (See Table S5 for Sample Identification)

Recovery studies were accomplished with a river water sample (R1.0, Table S6) spiked with 1.0 × 10^–5^ (R1.1) and 5.0 × 10^–5^ (R1.2) mol L^–1^ NH_4_^+^ concentrations. Each sample was analyzed in triplicate using both the potentiometric and conductivity detectors (Table 1 and Table S6). The recovery percentages were acceptable for both detectors, showing the appropriate accuracy of the potentiometry–IC methodology.

**Table 1:** Quantification of Ammonium Levels in Different Natural Water Samples Using the Potentiometry–IC and Conductivity–IC Setups^a^

	Potentiometry–IC		Conductivity–IC		
Sample	μmol L^–1^	RSD	μmol L^–1^	RSD	%Diff
R1.0	5.5	2.3	4.2	7.9	31
R1.1	13.0	2.8	14.0	1.2	7
R1.2	56.5	1.8	57.0	0.9	1
R2	2.6	8.4	ND		
R3	5.2	5.3	6.7	8.4	22
R4	4.7	3.1	ND		
L1	3.6	9.8	ND		
L2	4.0	7.1	ND		
L3	12.5	3.6	12.8	7.5	2
S1	4.2	8.3	ND		
S2	9.9	3.7	8.9	9.3	6
S3	4.0	6.7	ND		
S4	23.7	2.6	22.9	7.4	3

a ND = nondetectable. %Diff = percentage of difference between the results provided by potentiometry and conductivity. RSD = relative standard deviation.

**Figure 5:**
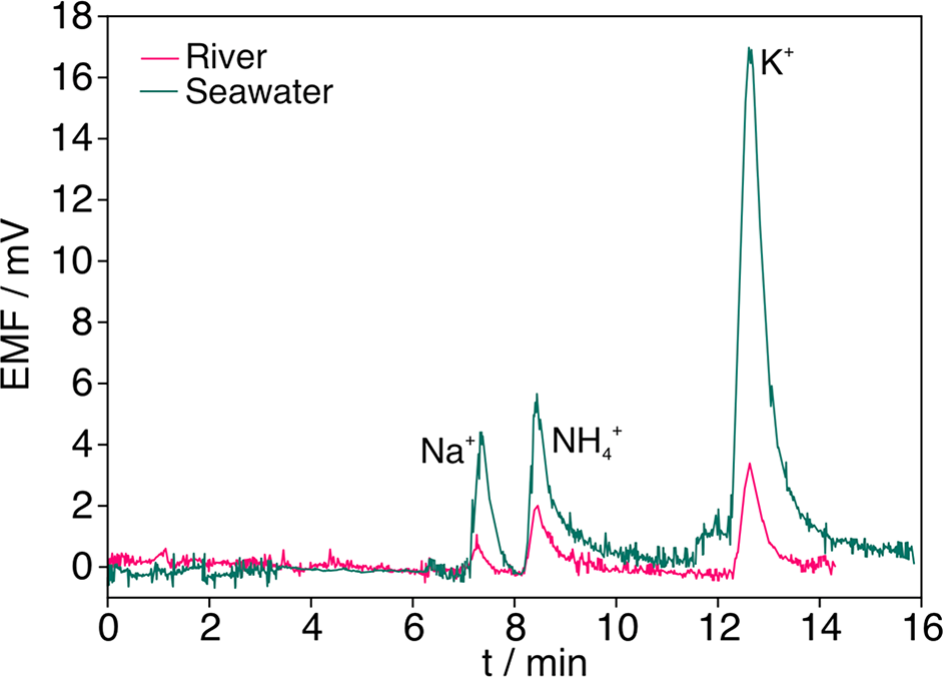
Chromatograms observed for two water samples: freshwater (river) and seawater.

Furthermore, the NH_4_^+^ content in ten water samples from different locations in Portugal, Sweden, and Spain (Table S5) was estimated. Some representative chromatograms are shown in Figure 5. Notably, the NH_4_^+^ peak was visible in all samples, approaching the level of noise (and potentiometric baseline) if the NH_4_^+^ content was close to the micromolar level. Peaks for K^+^ and Na^+^ were also observed in all the tested samples, presenting higher levels in the seawater samples. Nevertheless, there was no detected overlap with the NH_4_^+^ peak that could adversely affect its detection. The quantification of NH_4_^+^ was possible in all samples using the potentiometric detector, whereas the conductivity detector only detected NH_4_^+^ concentrations higher than 5 × 10^–6^ mol L^–1^. In those samples where a comparison was feasible, the results from both detectors agreed better at higher NH_4_^+^ concentrations. Overall, the potentiometric cell could detect and quantify lower NH_4_^+^ concentrations than the conductivity detector. The RSDs provided by both techniques were similar and always <10%.

**Figure 6:**
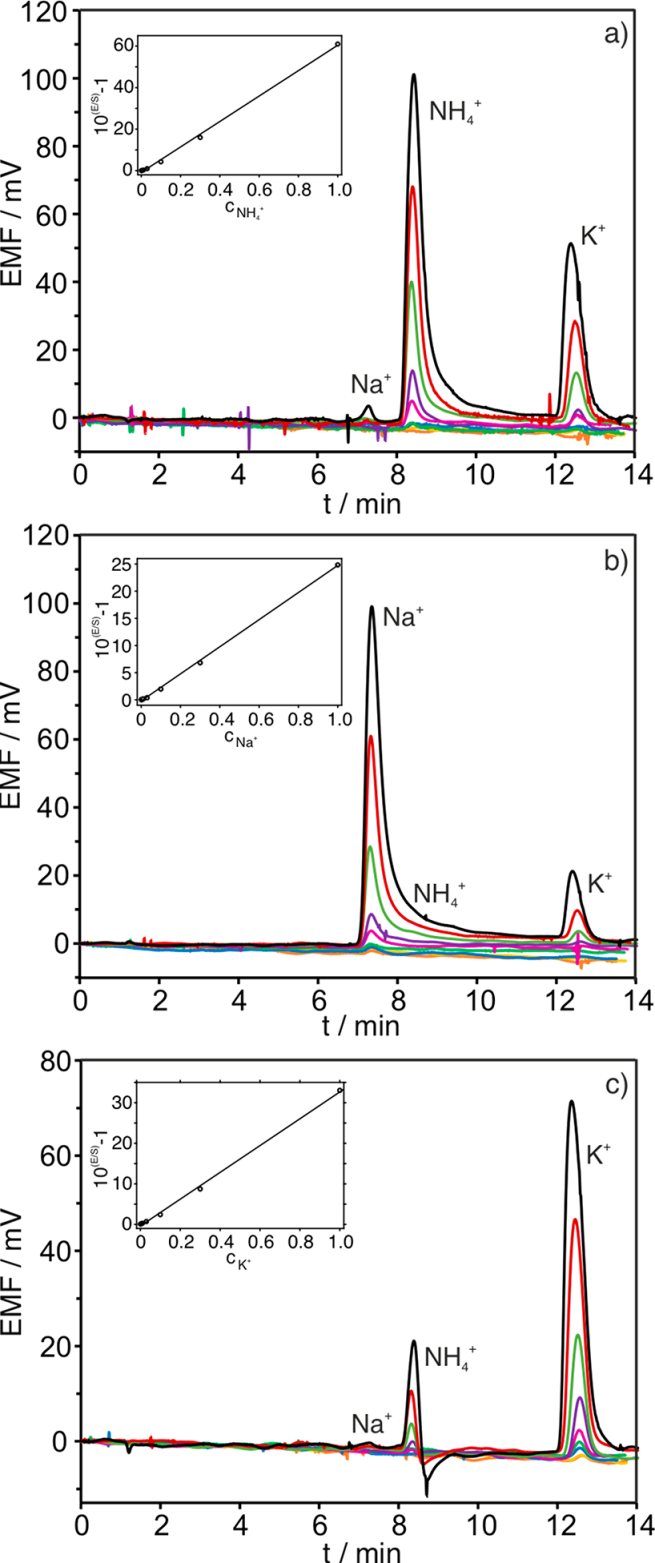
Chromatograms at increasing NH_4_^+^, Na^+^, and K^+^ activity provided by the (a) ammonium-selective electrode, (b) sodium-selective electrode, and (c) potassium-selective electrode. Insets: Linearized calibration graphs (2.5 × 10^–3^ mol L^–1^ nitric acid, 10 μL sample volume, flow rate of 0.9 mL min^–1^).

An interesting advantage of the potentiometric detector developed here is the possibility of simultaneously combining ISEs that are selective for different ions in the same cell for multi-ion detection. Accordingly, NH_4_^+^-, Na^+^-, and K^+^-selective electrodes were incorporated into the potentiometric cell as a preliminary proof-of-concept. The chromatograms and the corresponding calibration graphs obtained by using solutions containing the three cations at the same (increasing) concentrations are displayed in Figure 6. As observed, all the cations can be selectively detected by the corresponding electrode with no interference from the other cations. In more detail, the Na^+^-selective electrode presented a negligible response for NH_4_^+^ and a very low response for K^+^, whereas the K^+^-selective electrode displayed a negligible response for Na^+^ and some response for NH_4_^+^.

Regarding the calibration graph, in the case of Na^+^ and K^+^ electrodes, the LRR of the typical potentiometry plot (*i.e.*, EMF versus logarithmic activity) was found to include the levels expected in water samples (0.1–100 mM), and hence, implementing the linearization approach is not necessary (see Figure S4 for the calibration graphs and Table S7 for the corresponding calibration parameters). However, aiming at only one type of calibration, we used the linearized version of the calibration graph (as described above) for each of the three cations (NH_4_^+^, Na^+^, and K^+^) with the corresponding electrode. The calibration parameters in the LRR are collected in Table S8. Linear regression lines were obtained for NH_4_^+^, Na^+^, and K^+^ in the concentration range from 10^–6^ to 10^–3^ with sensitivities of 61.2 ± 2.5 (*R*^2^ = 0.9983), 24.9 ± 0.4 (*R*^2^ = 0.9979), and 33.1 ± 0.6 (*R*^2^ = 0.9973), respectively. The LOD and LOQ were calculated as the concentration corresponding to a signal-to-noise ratio of three and ten times, respectively, producing values of 3.0 × 10^–7^ (LOD) and 1.0 × 10^–6^ (LOQ) mol L^–1^ for the three cations.

The river samples R1.0, R1.1, and R1.2 were analyzed with the multi-cation potentiometric cell, revealing appropriate recovery percentages ranging from 81 to 102% for the potentiometric detector and from 73 to 140% for the conductivity one (Table 2). Considering the results provided by the conductivity detector, an excellent agreement was found, with a percentage of difference always lower than 18%, except for the R1.0 sample, which presented a 26% difference (Table 2). These results confirmed the great potential of the potentiometry–IC tandem for multi-ion detection.

**Table 2:** Quantification of Ammonium, Sodium, and Potassium Levels in Different Natural Water Samples Using the Potentiometry–IC and Conductivity–IC Setups^a^

		Potentiometry–IC			Conductivity–IC			
Sample	Cation	μmol L^–1^	RSD	%Rec	μmol L^–1^	RSD	%Rec	%Diff
R1.0	NH_4_^+^	4.1	2.6		5.2	8.8		26
	Na^+^	140.3	0.4		148.4	1.0		6
	K^+^	13.2	3.6		12.6	2.9		5
R1.1	NH_4_^+^	12.2	3.6	82	14.4	4.4	92	18
	Na^+^	149.8	3.1	95	162.4	1.3	140	8
	K^+^	21.3	6.1	81	19.9	5.0	73	7
R1.2	NH_4_^+^	47.8	3.6	88	53.8	6.7	97	12
	Na^+^	191.1	4.0	102	206.3	2.5	116	8
	K^+^	57.6	4.1	89	65.6	8.1	106	14

a %Rec = percentage of recovery. %Diff = percentage of difference between the results provided by potentiometry and conductivity.

### Analysis of the Developed Tandem Potentiometry–Ion Chromatography Method with Respect to the State-of-the-Art

To the best of our knowledge, the first attempts to coupling potentiometry to IC for (multi)ion detection used inner-filing solution-type ISEs.^23−26^ While the design of the electrode hindered the miniaturization of the entire system, these works demonstrated the great potential of potentiometry to detect ions after chromatographic separation, as it was sometimes superior to the analytical performance of the conductivity detector.^27,28^ It is worth mentioning that three main strategies have been followed in terms of the membrane composition: (i) a membrane containing a single ionophore that is selective for only one ion; (ii) a multi-ionophore membrane that is selective for at least two ions; and (iii) a membrane with a nonselective profile.^23−26,29^

The concept of a multi-ionophore membrane was translated to the solid-contact electrode type by Lee et al., who demonstrated the simultaneous analysis of K^+^, NH_4_^+^, Na^+^, and Ca^2+^ with a membrane containing four ionophores, one for each cation.^30^ However, the separation between NH_4_^+^, Na^+^, and K^+^ peaks was incomplete, likely due to the complex cross-selectivity profile normally found for this type of membrane. For NH_4_^+^ in particular, the observed LOD was close to 10^–5^ M, which is more than one order of concentration higher than that presented in this paper. Notably, NH_4_^+^ detection in real samples was not demonstrated.

Five years later, Isildak et al. presented tubular-shaped membrane electrodes with nonselective profiles for either cation or anion detection after chromatographic separation.^27,31^ Their cation detector was able to identify Na^+^, NH_4_^+^, K^+^, Rb^+^, Cs^+^, and Tl^+^ with well-separated peaks but with what appears to be a continuously drifting baseline, statement made based on the reported figures. The electrode was used in conjunction with a double-junction calomel reference electrode, which was not a solid-contact type because this technology was not yet common. For NH_4_^+^, the LOD was ca. 6 μM, which is again higher than that achieved in this paper and hence unsuitable for NH_4_^+^ detection in any water sample. The NH_4_^+^ concentration in two water samples (river and seawater) was calculated to be close to 20 μM, while the cation was not detectable in a tap water sample. Unfortunately, the results in real water samples were not validated with parallel measurements using a gold standard technique.

Shen et al. have reported chromatographic separation coupled to a solid-contact sensor array (*i.e.*, conducting wires covered by the membrane) in which each electrode was selective for one cation: Na^+^, NH_4_^+^, K^+^, Mg^2+^, and Ca^2+^.^32,33^ The authors achieved a constant baseline without any noticeable drift by including a 1 μM concentration of each cation in the eluent solution (*i.e.*, the background for the potentiometric measurements). The sensors presented LRRs from 0.05 to 1 mM. The ammonium content in some synthetic samples and a hydroponic solution was analyzed. However, the LRR did not allow the detection of NH_4_^+^ in a series of commercial and natural water samples.

The most recent paper reporting on NH_4_^+^ detection in mixtures dates from 2016 and is based on a solid-contact electrode (copper wire) modified with a carbon composite containing the membrane components.^34^ In this case, the membrane presented a nonselective profile, and after chromatographic separation, the NH_4_^+^ and K^+^ peaks appear with a high degree of overlap; thus, the LRR ranged between 5 × 10^–5^ and 10^–2^ M, which is not suitable for NH_4_^+^ detection in water samples. However, it is suitable for K^+^ detection.

Overall, the potentiometry–IC approach developed in the present paper is proposed within a state-of-the-art context that lacks a functional solution to detect NH_4_^+^ together with other cations in any water sample. The technology put forward regarding the potentiometry–chromatography system that can additionally include a conductivity detector is a unique method to validate all the measurements and demonstrates the advantages of the potentiometric readout over conductometry. The performance of the potentiometric sensors used to detect NH_4_^+^ (but also K^+^ and Na^+^) is superior to other sensors reported at the time of writing. Importantly, the potentiometric cell presents huge versatility for the detection of any ion.

## Conclusions

We demonstrate the necessity of the tandem potentiometry–IC system together with the linearization of the potentiometric response to address NH_4_^+^ detection at micromolar levels in any water sample where other cations (such as K^+^ and Na^+^) are present at higher levels. To pursue the decentralization of such analytical determination in diverse water environments, a miniaturized potentiometric detector based on a multielectrode flow cell was fabricated and characterized to provide the best analytical performances after in-line coupling with chromatographic separation. Advantageously, the potentiometric detector can be equipped with three similar ISEs to obtain reproducibility in the NH_4_^+^ quantification; or three electrodes, each selective for different ions, for multi-ion detection within the same sample. Preliminary results revealed the accurate and simultaneous detection of NH_4_^+^, Na^+^, and K^+^ in river samples. Soon, the implementation of the potentiometry–IC analytical concept in true *in situ* measurements is foreseen *via* potentiometric-ion-chromatography-on-a-chip technology. Simultaneous multi-ion detection is expected to be feasible in any kind of water (even such a complex matrix as seawater) and with high temporal and spatial resolution, thanks to further implementation in water platforms considering microfluidics.

## Supporting Information Available

Experimental section; tables with parameters from the chromatograms and calibration graphs; real samples (PDF)
