# Supplementary material for: Addressing the Detection of Ammonium Ion in Environmental Water Samples via Tandem Potentiometry–Ion Chromatography
Source: ACS Meas Sci Au. 2022 Jan 20;2(3):199–207. doi: 10.1021/acsmeasuresciau.1c00056 (PMC9204817; doi:10.1021/acsmeasuresciau.1c00056)
Supplement: Supplementary file 1 — tg1c00056_si_001.pdf [file tg1c00056_si_001.pdf]

Supporting information for:

Addressing the Detection of Ammonium Ion in Environmental Water Samples via Tandem Potentiometry–Ion Chromatography

Renato L. Gil[1], Célia G. Amorim[1] and Maria Cuartero[*,2]

[1]LAQV-REQUIMTE, Department of Chemical Sciences, Faculty of Pharmacy, University of Porto, Jorge Viterbo Ferreira, 228, 4050-313, Porto, Portugal.
[2] Department of Chemistry, School of Engineering Sciences in Chemistry, Biotechnology and Health, KTH Royal Institute of Technology, Teknikringen 30, SE-100 44, Stockholm, Sweden.

CORRESPONDING AUTHOR (*): M. Cuartero (mariacb@kth.se)

KEYWORDS. *Ammonium; Potentiometry; Ion-selective electrodes; Ion-chromatography; Environmental water samples; Tandem Analytical Technique.*

Experimental Section	…………………………………………………..	S2
Tables	…………………………………………………..	S4
Figures	…………………………………………………..	S7
References	…………………………………………………..	S10

Experimental Section

Reagents, materials and instrumentation. Ammonium ionophore I (Nonactin), Sodium ionophore X (4-tert-Butylcalix[4]arene-tetraacetic acid tetraethyl ester), Potassium ionophore I (valinomycin), sodium tetrakis[3,5-bis(trifluoromethyl)phenyl]borate (NaTFPB) and carboxylic acid functionalized multiwalled carbon nanotubes (MWCNTs) were purchased in selectophore grade from Sigma-Aldrich. High molecular weight poly(vinyl chloride) (PVC), polyvinyl butyral (PVB), dioctylsebacate (DOS), tetrahydrofuran (THF), ammonium chloride, magnesium chloride, calcium chloride, lithium chloride, sodium chloride, potassium chloride (KCl), and nitric acid (69%, HNO3) were obtained from Sigma Aldrich.

All solutions were prepared by dissolving the appropriate salts in 18.2 MΩ cm^{-1} doubly deionized water (Milli-Q water systems, Merck Millipore). The mobile phase in the IC system consisted of 2.5 mM nitric acid solution prepared by dilution of the corresponding concentrated form. Natural water samples (river, lake, and seawater) from Portugal, Sweden and Spain were collected at the surface of the corresponding aquatic resource with a falcon tube. The samples were filtered through 0.45 µm pore-size filters coupled to syringes and then stored in the fridge at 4 °C.

The cocktail for the NH_4^+ ISM was obtained by dissolving 1.0 mg of ammonium ionophore I (nonactin), 0.5 mg of NaTFPB, 66.5 mg of DOS, and 32.0 mg of PVC in 1 mL of THF. For the Na^+ ISM, 1.2 mg of sodium ionophore X, 0.5 mg NaTFPB, 66.0 mg DOS, and 33.0 mg of PVC were dissolved in 1 mL of THF. In the case of the K^+ ISM, 1.3 mg of potassium ionophore I, 0.5 mg of NaTFPB, 66.0 mg of DOS, and 33.0 mg of PVC were dissolved in 1 mL of THF. The cocktail for the reference membrane (RM) was prepared by dissolving 50 mg of sodium chloride and 78 mg of PVB in 1 mL of methanol.

The potential of the developed ISEs was measured with a high input impedance ($10^{15}\Omega$) EMF16 multichannel data acquisition device (Lawson Laboratories, Inc.) against either a double-junction Ag/AgCl/3M KCl/1M LiOAc reference electrode (6.0726.100, Metrohm Nordic, Sweden) or the hand-made solid-state reference electrode.

Before its coupling to the IC system, an ISMATEC peristaltic pump (Model IPC N-4 ISM 935) and PTFE tubing (L × O.D. × I.D. = 300 mm × 1/16 in. × 100 µm, Supelco) were used for the analytical characterization of the potentiometric cell.

Preparation of the ion-selective electrodes and reference electrode bodies. Miniaturized handmade glassy carbon electrodes (**Figure 1a**) were fabricated by gluing a glassy carbon rod (I.D. = 1.98 mm and 20 mm long, SIGRADUR®) inside a PEEK tube (Supelco) and maintaining a length of 5 mm of the rod outside the PEEK tube to make the electrical connections.[1] The surface of the carbon rod inside the PEEK was polished first with sandpaper (301D P400) until a flat surface was reached and then with alpha-alumina (0.5 microns) to reach a specular brightness of the glassy carbon material.

Potentiometric measurements. All experiments were carried out at room temperature of 22±1°C. To calibrate each ammonium-selective electrode, the dynamic potentiometric response was obtained at increasing concentrations of NH_4^+ in the sample solution and then, the corresponding logarithmic activities *(a_i)* were plotted versus the steady-state potential. Activity coefficients were calculated based on the Debye-Hückel theory to transform concentrations into activities and viceversa.[1-3] The data were fitted to the Nernst equation to obtain the sensitivity (slope) and intercept (i.e., whether the date was not

treated under the linearization protocol).[4] Unless otherwise indicated, the limit of detection (LOD) of the potentiometric electrodes was calculated as the activity related to the cross point between the extrapolation of the lines defining the nonresponsive range and linear-response range of the electrode.[5]

Tables

Table S1. Signal output provided by the potentiometry–IC and conductivity–IC setups.

Cation	Concentration (mol L^{-1})	Potentiometry–IC		Conductivity–IC		
		Rt (min)	Peak Height (mV)	Rt (min)	Peak Height (µS cm^{-1})	Peak Area (µS cm^{-1} min^{-1})
Li^{+}	1.0x10^{-3}	5.9	0.33	5.8	14.00	2.26
Na^{+}	1.0x10^{-3}	8.4	2.35	8.4	11.81	2.55
NH_4^{+}	1.0x10^{-3}	9.7	80.89	9.6	10.36	2.33
K^{+}	1.0x10^{-3}	14.5	44.38	14.4	6.60	2.36

Rt: Retention time

Table S2. Signal output of increasing NH_4^{+} concentration provided by the potentiometry–IC and conductivity–IC setups (concentration of Li^{+}, Na^{+}, and K^{+} was fixed at 1.0x10^{-3} mol L^{-1}).

Cation	Concentration (mol L^{-1})	Potentiometry–IC		Conductivity–IC		
		Rt (min)	Peak Height (mV)	Rt (min)	Peak Height (µS cm^{-1})	Peak Area (µS cm^{-1} min^{-1})
NH_4^{+}	1.0x10^{-6}	9.8	1.65	9.6	0.03	0.01
	1.0x10^{-5}	9.6	5.13	9.6	0.12	0.03
	1.0x10^{-4}	9.7	30.23	9.6	1.34	0.29
	1.0x10^{-3}	9.7	80.89	9.7	10.36	2.33

Rt: Retention time

Table S3. Chromatographic parameters obtained for the ammonium peak using the potentiometry–IC setup at different injection volumes.

	Injection volume					
	10 µL			20 µL		
NH_4^{+} activity	1.0x10^{-5}	1.0x10^{-4}	1.0x10^{-3}	1.0x10^{-5}	1.0x10^{-4}	1.0x10^{-3}
**Rt**	9.7	9.7	9.8	9.9	9.9	10.3
**k**	6.5	6.5	6.5	6.6	6.6	6.9
**Peak width**	1.3	2.1	2.4	2.0	2.7	4.2
**As**	2.5	3.5	3.3	2.7	2.9	2.3
**N**	3581	2389	1663	1780	1225	434

Rt: retention time in minutes; k: capacity factor; Peak width was measured as the difference between the time in minutes at 5% of the peak maximum; As: peak symmetry; N: number of theoretical plates.

Table S4. Chromatographic parameters obtained for the ammonium peak using the potentiometry–IC setup at different flow rates (average of 1.0x10^{-3}, 1.0x10^{-4} and 1.0x10^{-5} NH_4^{+} activity).

	Flow rate (mL min^{-1})

	0.5	0.7	0.9
**Rt**	15.4	11.0	8.5
**k**	10.8	7.5	5.5
**Peak width**	2.9	2.2	1.7
**As**	2.5	2.6	3.1
**N**	1584	2254	3040

Rt: retention time in minutes; k: capacity factor; Peak width was measured as the difference between the time in minutes at 5% of the peak maximum; As: peak symmetry; N: number of theoretical plates.

Table S5. Identification of environmental water samples and approximate location.

Sample	Type	Country	Geographic coordinates
R1	River	Portugal	41°35'45.6"N 8°27'49.7"W
R2	River	Sweden	59°51'48.3"N 17°37'51.0"E
R3	River	Spain	37°59'25.09"N 1°5'18.76"W
R4	River	Spain	37°59'4.14"N 1°10'18.50"W
L1	Lake	Sweden	59°18'43.6"N 18°00'58.6"E
L2	Lake	Spain	38°02'07.0"N 1°40'18.3"W
L3	Lake	Spain	38°13'14.8"N 1°58'28.4"W
S1	Seawater	Sweden	59°18'54.0"N 18°01'28.5"E
S2	Seawater	Sweden	59°19'40.7"N 18°03'22.3"E
S3	Seawater	Sweden	59°21'36.0"N 18°05'24.8"E
S4	Seawater	Spain	38°50'05.1"N 0°07'54.6"E

Table S6. Recovery values of the potentiometry–IC and conductivity–IC setups for ammonium determination in a spiked river sample (n=3).

Sample	Added (µM)	Potentiometry–IC			Conductivity–IC		
		Found (µM)	RSD	% Recovery[a]	Found (µM)	RSD	% Recovery[a]
**R1.0**	0	5.5		–	4.2		–
**R1.1**	10	13.0	2.8	75.3	14.0	1.2	98.5
**R1.2**	50	56.5	1.8	102.1	57.0	0.9	105.5

[a] The recovery percentage was calculated according to the formula:

$$Recovery\ (\%) = \frac{NH_4^+{}_{Found} - NH_4^+{}_{Initial}}{NH_4^+{}_{Added}}\ x\ 100\%$$

Table S7. Linear fittings (potential versus logarithmic activity) within the LRR for NH_4^+-, Na^+- and K^+-selective electrodes incorporated in the microfluidic cell in the potentiometry–IC setup (n=2). Coefficient of variations in % are provided in parenthesis.

	Slope (mV dec^{-1})	Intercept (mV)	R^2	LRR (activity)	LOD (activity)
**$NH4^+$ - ISE**	57.3 ± 0.3 (0.4)	272.5 ± 0.3 (0.1)	0.9966 ± 0.0006 (0.1)	3.0x10^{-5} to 1.0x10^{-3}	(1.9 ± 0.0)x10^{-5} (2.7)

**Na^+ - ISE**	71.1 ± 0.5 (0.7)	312.9 ± 2.8 (0.9)	0.9984 ± 0.0004 (0.0)	1.0x10^{-4} to 1.0x10^{-3}	(4.2 ± 0.3)x10^{-5} (7.0)
**K^+ - ISE**	48.7 ± 0.6 (1.3)	219.9 ± 3.6 (1.7)	0.9947 ± 0.0036 (0.4)	1.0x10^{-4} to 1.0x10^{-3}	(3.5 ± 0.2)x10^{-5} (4.6)

LRR: Linear response range; LOD: Limit of detection.

Table S8. Calibration parameters for NH_4^+-, Na^+- and K^+-selective electrodes incorporated in the microfluidic cell in the potentiometry–IC setup (n=3) using the linearized approach for the calibration procedure. Coefficient of variations in % are provided in parenthesis.

	**Slope (mM^{-1})**	**Intercept (-)**	**R^2**	**LRR (mol L^{-1})**	**LOQ (mol L^{-1})**	**LOD (mol L^{-1})**
**$NH4^+$ - ISE**	61.2 ± 2.5 (4.1)	-0.8 ± 0.0 (3.8)	0.9983 ± 0.0000 (0.0)	1.0x10^{-6} to 1.0x10^{-3}	1.0x10^{-6}	3.0x10^{-7}
**Na^+ - ISE**	24.9 ± 0.4 (1.7)	-0.3 ± 0.0 (6.6)	0.9979 ± 0.0002 (0.0)	1.0x10^{-6} to 1.0x10^{-3}	1.0x10^{-6}	3.0x10^{-7}
**K^+ - ISE**	33.1 ± 0.6 (1.9)	-0.4 ± 0.1 (26.4)	0.9973 ± 0.0012 (0.1)	1.0x10^{-6} to 1.0x10^{-3}	1.0x10^{-6}	3.0x10^{-7}

LRR: Linear response range; LOQ: Limit of quantification; LOD: Limit of detection

Figures

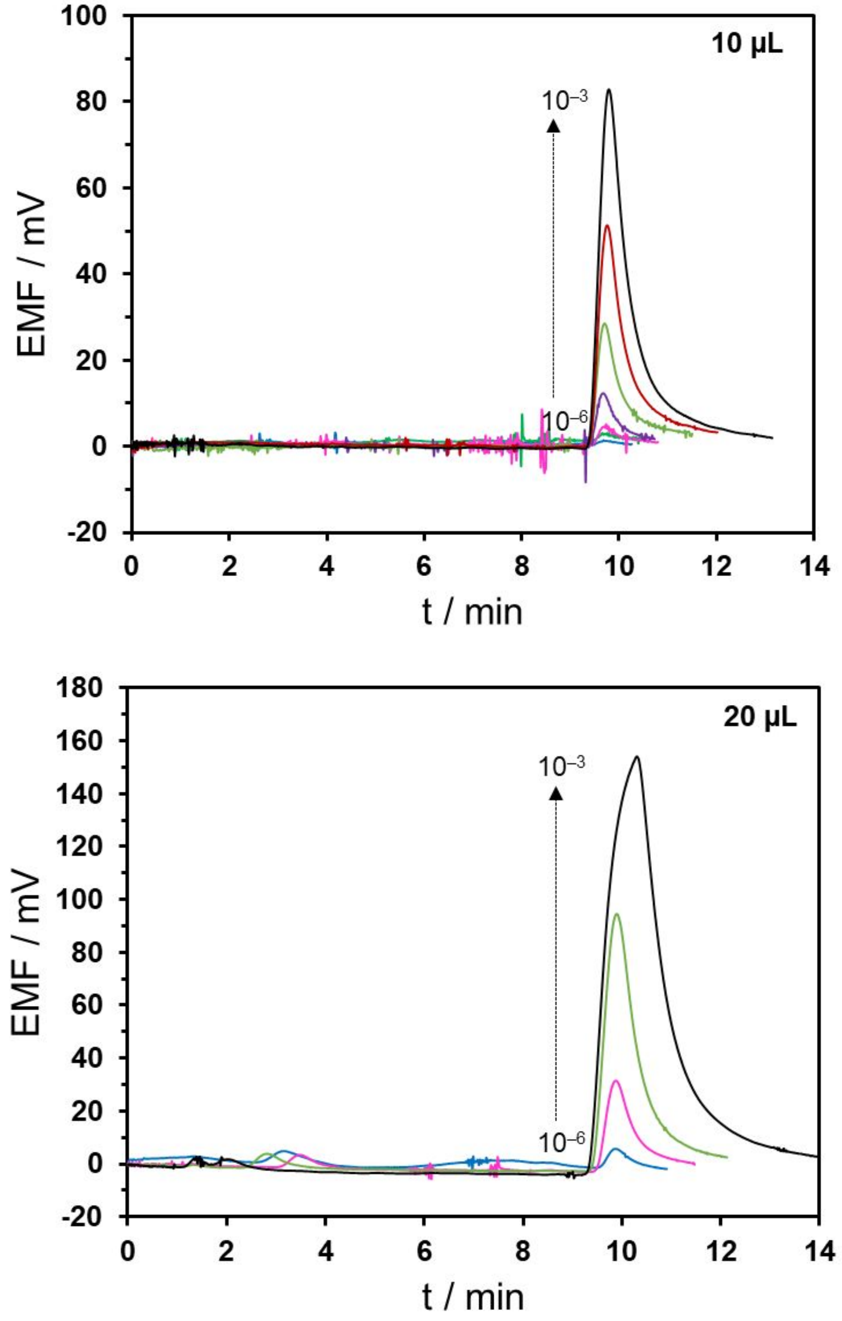

Figure S1. Potentiometric chromatograms with 10 and 20 µL injected sample volume at increasing NH_4^+ activity ($2.5x10^{-3}$ mol L^{-1} nitric acid, 0.9 mL min^{-1}).

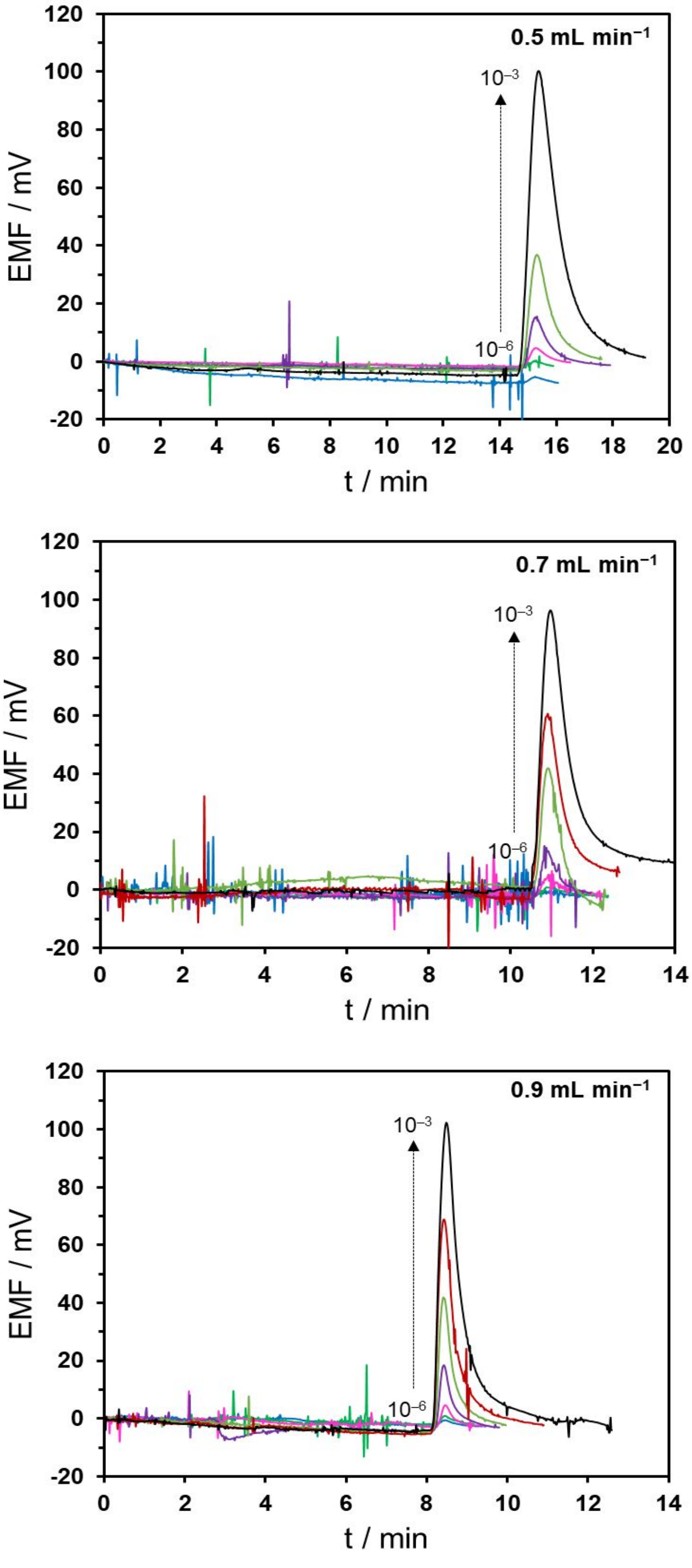

Figure S2. Potentiometric chromatograms with 0.5, 0.7 and 0.9 mL min^{-1} at increasing NH_4^+ activity (2.5×10^{-3} mol L^{-1} nitric acid, 10 µL volume).

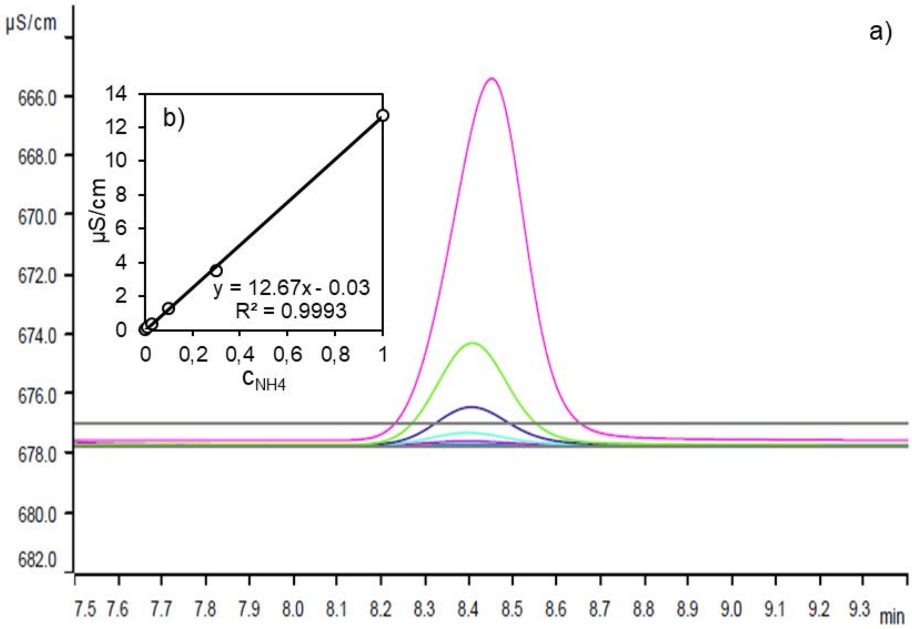

Figure S3. (a) Conductimetric chromatograms at increasing NH_4^+ activity. (b) Corresponding calibration graph ($2.5x10^{-3}$ mol L^{-1} nitric acid, 10 µL sample volume, flow rate of 0.9 mL min^{-1}).

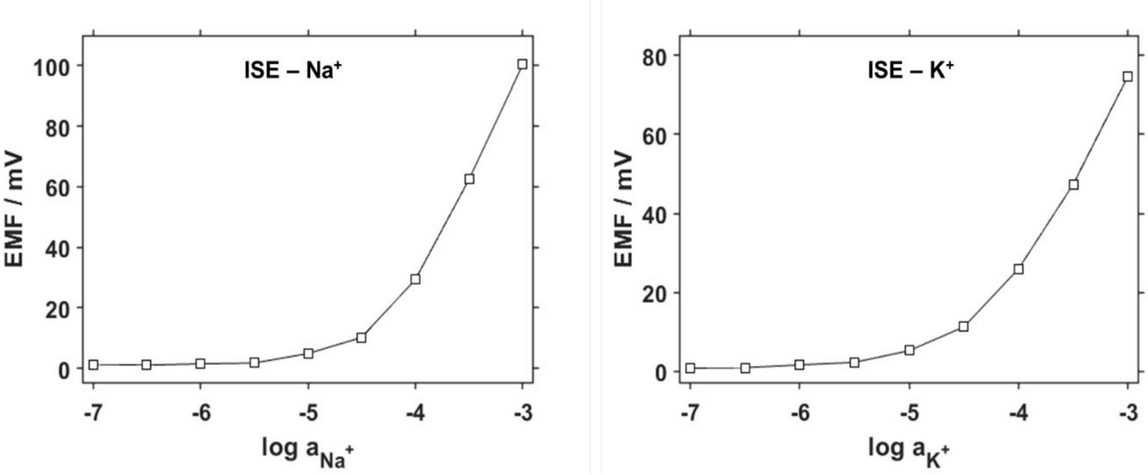

Figure S4. Calibration graphs (potential versus logarithmic activity) for Na^+- and K^+-selective electrodes incorporated in the microfluidic cell in the potentiometry–IC setup at increasing Na^+ and K^+ activity ($2.5x10^{-3}$ mol L^{-1} nitric acid, 10 µL sample volume, flow rate of 0.9 mL min^{-1}).

References

(1) Cuartero, M.; Pankratova, N.; Cherubini, T.; Crespo, G. A.; Massa, F.; Confalonieri, F.; Bakker, E. In situ detection of species relevant to the carbon cycle in seawater with submersible potentiometric probes. *Environ. Sci. Tech. Let.* **2017**, 4, 410-415.
(2) Meier, P. C. Two-parameter debye-hückel approximation for the evaluation of mean activity coefficients of 109 electrolytes. *Anal. Chim. Acta* **1982**, 136, 363-368.
(3) Pankratova, N.; Crespo, G. A.; Afshar, M. G.; Crespi, M. C.; Jeanneret, S.; Cherubini, T.; Tercier-Waeber, M. L.; Pomati, F.; Bakker, E. Potentiometric sensing array for monitoring aquatic systems. *Environ. Sci.: Process. Impacts* **2015**, 17, 906-914.
(4) Bakker, E.; Pretsch, E. Modern potentiometry. *Angew. Chem. Int. Ed. Engl.* **2007**, 46, 5660-5668.
(5) Richard P. Buch, E. L. Recomendations for nomenclature of ion-selective electrodes (IUPAC Recommendations 1994). *Pure Appl. Chem.* **1994**, 66, 2527-2536.
